# Supplementary material for: Genetic dissection of grain iron concentration in hexaploid wheat (Triticum aestivum L.) using a genome-wide association analysis method
Source: PeerJ. 2022 Jul 22;10:e13625. doi: 10.7717/peerj.13625 (PMC9310890; doi:10.7717/peerj.13625)
Supplement: Supplemental Information 1 — Edited Certificate for Manuscript [file peerj-10-13625-s001.pdf]

Certificate of English Language Editing

Manuscript Title:

Genetic Dissection of Grain Iron Concentration in Hexaploid Wheat (Triticum aestivum L.) Using a Genome-Wide Association Analysis Method

Date of Revision:

May 12, 2022

Abstract:
Iron (Fe) is an essential micronutrient of the body. Low concentrations of bioavailable Fe in staple food result in micronutrient malnutrition. Wheat (Triticum aestivum L.) is the most important global food crop and thus has become an important source of iron for people. Breeding nutritious wheat with high grain Fe content has become an effective means of alleviating malnutrition. Understanding the genetic basis of micronutrient concentration in wheat grains may provide useful information for breeding for high Fe varieties through marker-assisted selection (MAS). Hence, in the present study, genome-wide association studies (GWAS) were conducted for grain Fe. An association panel of 207 accessions was genotyped using a 660K SNP array and phenotyped for grain Fe content at three locations. The genotypic and phenotypic data obtained thus were used for GWAS. A total of 911 SNPs were significantly associated with grain Fe concentrations. These SNPs were distributed on all 21 wheat chromosomes, and each SNP explained 5.79%–25.31% of the phenotypic variations. Notably, the two significant SNPs...

This document certifies that the manuscript listed above was copy edited for English language by LetPub, with regard to grammar, punctuation, spelling, and clarity. All of our language editors are native English speakers with long-term experience in editing scientific and technical manuscripts. We are committed to leveling the playing field for researchers whose native language is not English.

- Documents receiving this certification should be regarded as having undergone professional editorial revision for English language before submission. However, the authors may accept or reject LetPub's suggestions and changes at their own discretion and LetPub does not have editorial control over the submitted documents.
- The language quality of the submitted document is the sole responsibility of the submitting authors subject to those authors' adherence to LetPub's revisions and instruction. LetPub's provision of service does not constitute a guarantee or endorsement of the authors' work herein.
- Neither the research content nor the authors' intended meaning were altered in any way during the editing process.
- If you have any questions or concerns about this edited document, please contact us at support@letpub.com

LetPub
Your Manuscript Editor

LetPub is an author service brand owned and operated by Accdon LLC. Headquartered in the Boston area, we are a full-spectrum author services company with a large team of US-based certified language and scientific editors, ISO 17001 accredited translators, and professional scientific illustrators and animators. We advocate ethical publication practices and are an official member of the Committee on Publication Ethics (COPE).

For more information about our company, services, and partnership programs, please visit www.letpub.com.
© 2022 Accdon, LLC. All Rights Reserved. Tel: 1-781-202-9968 Email: info@accdon.com Address: 400 Fifth Ave, Suite 530, Waltham, MA 02451, United States
